# Systemic Immune-Inflammation Index Predicts Prognosis of Patients with Esophageal Squamous Cell Carcinoma: A Propensity Score-matched Analysis

**DOI:** 10.1038/srep39482

**Published:** 2016-12-21

**Authors:** Yiting Geng, Yingjie Shao, Danxia Zhu, Xiao Zheng, Qi Zhou, Wenjie Zhou, Xuefeng Ni, Changping Wu, Jingting Jiang

**Affiliations:** 1 Department of Oncology, The Third Affiliated Hospital of Soochow University, 185 Juqian Street, Changzhou 213003, P.R. China; 2 Department of Radiation Oncology, The Third Affiliated Hospital of Soochow University, 185 Juqian Street, Changzhou 213003, P.R. China; 3 Department of Tumor Biological Treatment, The Third Affiliated Hospital of Soochow University, 185 Juqian Street, Changzhou 213003, P.R. China

## Abstract

Systemic immune-inflammation index (SII), based on peripheral lymphocyte, neutrophil, and platelet counts, was recently investigated as a prognostic marker in several tumors. However, SII has not been reported in esophageal squamous cell carcinoma (ESCC). We evaluated the prognostic value of the SII in 916 patients with ESCC who underwent radical surgery. Univariate and multivariate analyses were calculated by the Cox proportional hazards regression model. The time-dependent receiver operating characteristics (ROC) curve was used to compare the discrimination ability for OS. PSM (propensity score matching) was carried out to imbalance the baseline characteristics. Our results showed that SII, PLR, NLR and MLR were all associated with OS in ESCC patients in the Kaplan-Meier survival analysis. However, only SII was an independent risk factor for OS (HR = 1.24, 95% CI 1.01–1.53, *P* = 0.042) among these systemic inflammation scores. The AUC for SII was bigger than PLR, NLR and MLR. In the PSM analysis, SII still remained an independent predictor for OS (HR = 1.30, CI 1.05–1.60, *P* = 0.018). SII is a novel, simple and inexpensive prognostic predictor for patients with ESCC undergoing radical esophagectomy. The prognostic value of SII is superior to PLR, NLR and MLR.

Esophageal cancer is a common cancer worldwide. In China, it is the 3th leading cancer in incidence and 4th in mortality^1^. In 2015, there were 477,900 new cases and 375,000 deaths of esophageal cancer in China^1^. Although the development of its multidisciplinary treatment, five-year overall survival (OS) for esophageal cancer is 15–35% and the prognosis remains poor^2^. The two main pathological subtypes of esophageal cancer are squamous cell carcinoma and adenocarcinoma. In China, esophageal squamous cell carcinoma (ESCC) accounts for over 90% of cases^3^. The 7th edition American Joint Committee on Cancer tumor-node-metastasis (AJCC TNM) staging system is used to distinguish the prognosis among different risk groups of ESCC patients. However, ESCC patients at the same TNM stage and received similar therapy usually had variable outcomes. Therefore, it is important to explore dependable prognostic factors.

In recent years, neutrophil, platelet and lymphocyte derived from the peripheral blood were significantly associated with tumor progression in various tumors^4^,^5^,^6^. Some indicators such as neutrophil lymphocyte ratio (NLR) based on neutrophil and lymphocyte, platelet lymphocyte ratio (PLR) based on platelet and lymphocyte, and monocyte lymphocyte ratio (MLR) have emerged as prognostic factors in many cancers, including ESCC^7^,^8^,^9^,^10^. These indicators only integrate two cells. Systemic immune-inflammation index (SII), based on peripheral lymphocyte, neutrophil, and platelet counts, was recently investigated as a prognostic marker in several tumors including hepatocellular carcinoma^11^,^12^, colorectal cancer^13^ and small cell lung cancer^14^. However, SII has not been reported in ESCC. In this study, we evaluated the prognostic value of SII in patients with ESCC who underwent radical surgery. We also explore whether SII has more advantages to predict the survival of ESCC population than NLR or PLR. To increase statistical power and to further elaborate on the possible prognostic impact of SII, both Cox’s proportional hazards model analysis as well as propensity score matching (PSM) were applied.

## Results

### Clinicopathological characteristics of Patient

There were 696 males (76.0%) and 220 females (24.0%) with an age range of 37–84 years (median 60.0 years), of which 46 patients were well differentiated, 450 patients were moderately differentiated, and 420 patients were poorly differentiated. According to the 7th AJCC standard, there were 168 patients at stage 0-I, 395 patients at stage II and 353 patients at stage III. Other clinicopathological features are shown in Table 1. The median OS was 42 months (range, 3 to 146 months) and the rate of 3- and 5-year OS was 52.5% and 44.2%, respectively. Patients with SII > 307 in complete datasets were more likely to be men (*P* = 0.022), poor differentiation (*P* = 0.004), advanced T stage (*P* < 0.001), advanced N stage (*P* < 0.001) and advanced AJCC TNM stage (*P* < 0.001) (Table 1). Patients with NLR > 1.7 showed the similar results. PLR > 120 was only associated with advanced AJCC TNM stage (*P* = 0.015). MLR was associated with sex (*P* < 0.001), T stage (*P* = 0.002) and AJCC TNM stage (*P* < 0.001) in complete datasets (Table 2).

### The prognostic significance of SII, NLR, PLR and MLR

**Figure 1:**
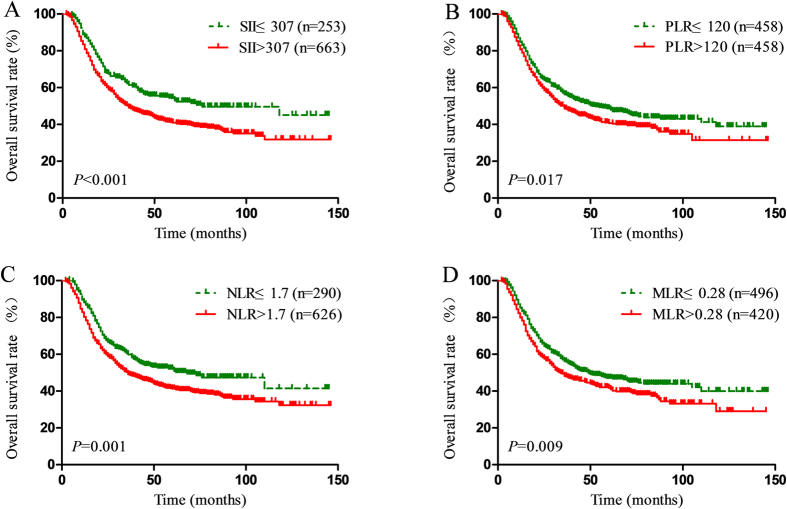
Kaplan–Meier survival curves for patients stratified based on (**A**) SII, (**B**) PLR, (**C**) NLR and (**D**) MLR in unmatched complete datasets.

**Figure 2:**
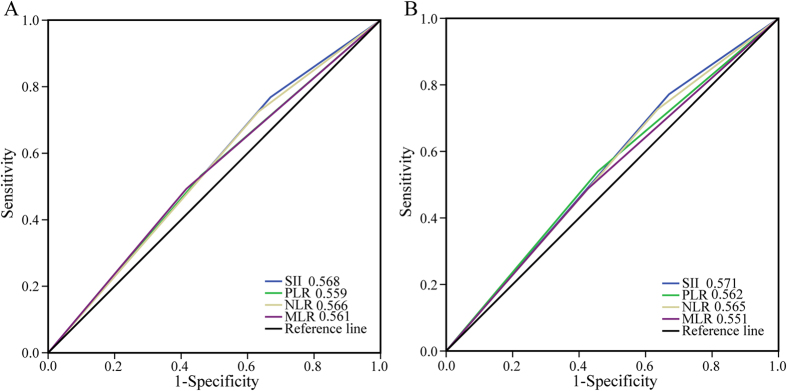
Predictive ability of the SII was compared with PLR, NLR and MLR by ROC curves in 3-years (**A**) and 5-years (**B**).

The Kaplan-Meier survival analysis showed that high SII, PLR, NLR and MLR scores were all associated with poor OS in ESCC patients (*P* < 0.001, *P* = 0.017, *P* = 0.001, *P* = 0.009, respectively) (Fig. 1). The median OS was 76 months for patients with SII ≤ 307 and 36 months for patients with SII > 307. In addition, patients with PLR ≤ 120 had a median OS of 53 months, whereas patients with PLR > 120 had a median OS of 36 months. Patients with NLR ≤ 1.7 had a median OS with 68 months, compared with 36 months for the patients with NLR > 1.7. Patients with MLR ≤ 0.28 had a median OS with 49 months, compared with 34 months for patients with MLR > 0.28. Based on the univariate analysis, sex, histological grade, T stage, N stage, SII, PLR, NLR and MLR were identified as the significant prognostic factors (Table 3). It was found that SII, PLR, NLR and MLR were highly correlated and had the same factors. There four separate multivariate models (SII, PLR, NLR and MLR) were run to avoid problems with the presence of multicollinearity. Multivariate analyses demonstrated that histological grade, T stage, N stage, and SII were independent risk factors for OS (Table 3). Among SII, NLR, PLR and MLR, only SII was an independent risk factor for OS (HR = 1.24, 95% CI 1.01–1.53, *P* = 0.042). In addition, the discrimination ability of SII, PLR, NLR and MLR was compared by the AUC for OS. The AUC for SII was bigger than SII, NLR, PLR and MLR for predicting survival in patients with ESCC in 3-years and 5-years (Fig. 2). It means SII is superior to NLR, PLR or MLR as a predictive factor in ESCC patients.

### Propensity score matching analysis

**Figure 3:**
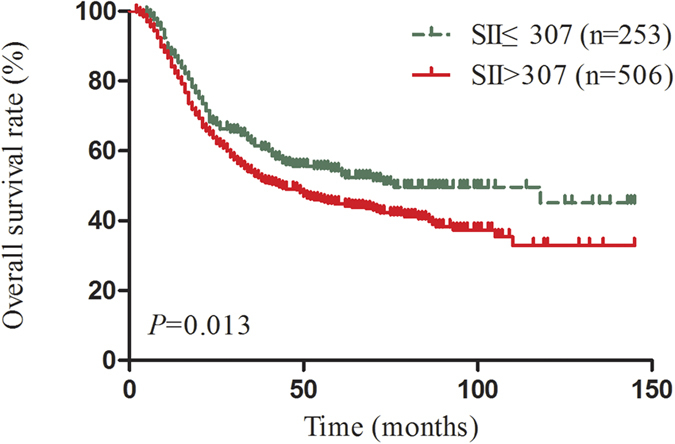
Kaplan-Meier-estimated overall survival distributions from matched datasets for SII ≤ 307 versus SII > 307.

Considered the sex, histological grade, T stage, N stage and AJCC TNM stage were imbalance between SII ≤ 307 and SII > 307 ESCC patients (Table 1), we applied a 1:2 PSM ratio to minimize these differences. In the PSM analysis, we selected 253 patients from SII ≤ 307 group with matched pairings of the 506 SII > 307 patients using a nearest-neighbour algorithm. These clinicopathological characteristics were balanced and evenly distributed between these groups (all *P* > 0.1) (Table 1). The Kaplan-Meier survival curves for the matched groups are shown in Fig. 3. In the matched 759 patients’ survival analysis, median OS was 76 months for SII ≤ 307 ESCC patients and 43 months for SII > 307 ESCC patients. The 5-year survival was 55.4% in SII ≤ 307 ESCC patients versus 44.9% in SII > 307 ESCC patients. In addition, the multivariate analyses showed SII still remained an independent predictor for OS (HR = 1.30, CI 1.05-1.60, *P* = 0.018) (Table 4).

## Discussion

Inflammation has been known as a hallmark feature of tumor^15^. The correlation between inflammation and tumor was first reported by Rudolf Virchow in 1863^16^. Recently, accumulating evidence has indicated that inflammation contributes to tumor development, progression and metastasis. Systemic inflammatory scores such as NLR, PLR and MLR have been found to be independent markers of prognosis in a variety of cancers, including ESCC^7^,^8^,^9^,^10^. A novel systemic inflammation score-SII, based on neutrophil, platelet, and lymphocyte counts, was shown to be an independent risk of recurrence and survival for hepatocellular carcinoma, small cell lung cancer, colorectal cancer and gastric cancer patients^11^,^12^,^13^,^14^,^17^. It was considered to be better than PLR and NLR, and was associated with higher circulating tumor cells (CTCs) levels. In the present study, SII was confirmed to be a novel independent predictor of survival for patients with resectable ESCC by a multivariable Cox regression analysis and PSM analysis. It was shown to be superior to NLR, PLR and MLR as a predictive factor in ESCC patients. Compared with other prognostic factors, the inflammation-based prognostic scores are simple, inexpensive and routinely performed in clinical practice. Meanwhile, SII based on standard laboratory measurements of total platelet, neutrophil, and lymphocyte counts is simple, inexpensive and routinely performed in clinical practice. Thus, there is a potential for SII to be used as a marker for prognosis and treatment response surveillance.

Several potential mechanisms may be used to explain the prognostic values of SII in tumor. Cancer-mediated myelopoiesis has been recognised in the promotion of tumor angiogenesis, cell invasion, and metastasis in recent years. In contrast with myelopoiesis during acute infection, stress, or trauma in which circulating immune cells are transient increase, cancer myelopoiesis is associated with persistence of immature myeloid cells^18^. Firstly, neutrophils are not released from the bone marrow until mature ordinarily, however, in the context of inflammation, they were triggered by secretion of cytokines and chemokines, such as interleukin-6 (IL-6), tumor necrosis factor (TNF) and myeloid growth factors^19^. These inflammatory mediators enhance the invasion, proliferation, and metastasis of cancer cell, aid cancer cells to evade immune surveillance, and induce the resistance to cytotoxic drugs^6^,^20^. The elevated neutrophils can also release plenty of nitric oxide, arginase, and reactive oxygen species (ROS), leading to T cell activation disorders^21^. Secondly, platelets can protect CTCs from shear stresses during circulation, induce epithelial-mesenchymal transition, and promote tumor cell extravasation to metastatic sites^4^,^22^. Meanwhile, platelets and neutrophils have been reported to promote adhesion and seeding of distant organ sites through secreting vascular endothelial growth factor (VEGF)^5^,^11^,^23^. Thirdly, lymphocytes can also secrete several cytokines, such as IFN-γ and TNF-α, to control tumor growth and improve prognosis of cancer patients^24^, and the decreased lymphocyte count and function will impair cancer immune surveillance and defense^6^,^24^.

Based on the above theory, SII should be a more objective marker that reflects the balance between host inflammatory and immune response status than all the other systemic inflammation index such as the PLR and NLR. In fact, our results confirmed that SII is indeed superior to PLR, NLR and MLR. In addition, many studies have confirmed that non-steroidal anti-inflammatory drugs (NSAIDs) are associated with improved survival outcomes in patients with cancer, including esophageal cancer^25^,^26^. The patients with ESCC who have a high SII maybe especially benefit from targeted anti-inflammatory with aspirin and non-steroidal anti-inflammatory drugs (NSAIDs).

Although our results demonstrated the prognostic value of SII in ESCC, there are still several limitations in this study. First, it should be noted that most patients with esophageal cancer in China are squamous cell carcinoma, while the most esophageal cancer is adenocarcinoma in western. Therefore, the prognostic significance of SII needs to be validated in patients with esophageal adenocarcinoma. Second, our study was a retrospective study, and there may be selection bias during retrospective data collection. However, we used PSM analysis which can minimize group differences in the baseline characteristics. Third, the majority (77.8%) of patients enrolled in this study had dissected lymph nodes with <15, so further studies for patients with adequate lymphadenectomy are needed to confirm our results. Fourth, our study is a single retrospective center research study. Thus, a multicenter collaborative prospective study is required to be further verified in a prospective, large-scale collaborative study.

In conclusion, SII is a novel independent prognostic predictor for patients with ESCC undergoing radical esophagectomy. The prognostic value of SII is superior to PLR, NLR and MLR. Based on simple and inexpensive standard laboratory measurements, SII will be a potential marker for ESCC prognosis and treatment response surveillance.

## Materials and Methods

### Patients

A retrospective analysis was conducted in patients who underwent radical esophagectomy at the Third Affiliated Hospital of Soochow University (Changzhou, China) from January 2002 to December 2012. The dissection area for lymphadenectomy was described as our previous article^27^. All patients received transthoracic radical esophagectomy with mediastinal and abdominal two-field lymphadenectomies. The scope of mediastinal lymphadenectomies included subcarinal, left and right bronchial, lower posterior mediastinum, pulmonary ligament, and paraesophageal and thoracic duct nodes. The scope of abdominal lymphadenectomies included the paracardial, lesser curvature, left gastric, common hepatic, celiac, and splenic nodes. The paratracheal and recurrent laryngeal nerve LNs were also dissected. Cervical lymphadenectomy was not conventionally performed, except for cases of suspicious cervical lymphadenopathy. The inclusion criteria were as follows: ESCC was confirmed by histopathology, R0 resection, no preoperative or postoperative radiotherapy and/or chemotherapy. At last, 916 patients were enrolled in the current study. The patient follow-up started from the date of surgery and continued up until December 2014 or patients’ death. All the patients received postoperative follow-up every 3 months within two years after the operation, and the median follow-up time was 39 months (3–146 months). This study was undertaken according to the Declaration of Helsinki and was approved by the Ethics Committee of Third Affiliated Hospital of Soochow University. Written informed consent was obtained from all participants.

Data on preoperative peripheral neutrophil, lymphocyte, and platelet counts were extracted from the medical records. The definitions of SII, NLR and PLR are described as follows: SII = platelet*neutrophil/lymphocyte; NLR = neutrophil/lymphocyte; PLR = platelet/lymphocyte. The optimal cutoff values including SII (SII ≤ 307, SII > 307), NLR (NLR ≤ 1.7, NLR > 1.7), PLR (PLR ≤ 120, PLR > 120) and MLR (MLR ≤ 0.28, NLR > 0.28) were determined by using X-tile software (http://www.tissuearray.org/rimmlab)^28^.

### Statistical Analysis

Statistical analysis was conducted with SPSS 22.0 (SPSS, Chicago, IL), Graphpad Prism 6.01 (La Jolla, CA, USA) and R software 3.2.5 (http://www.r-project.org/) with MatchIt packages. The correlations between the inflammation-based prognostic scores and clinicopathological characteristics were analyzed by the χ^2^ test. Correlation analysis is using Person’s correlation test. Survival curves were plotted using the Kaplan-Meier method and compared using the log-rank test. Univariate and multivariate analyses were calculated by the Cox proportional hazards regression model. The time-dependent receiver operating characteristics (ROC) curve was used to compare the discrimination ability for OS. PSM was carried out because of imbalance in the baseline characteristics. PSM was done with a nearest-neighbour matching algorithm, allowing a maximum tolerated difference between propensity scores less than 30% of the propensity score SD. A *P* value less than 0.05 was considered to be statistically significant unless otherwise specified.

## Additional Information

**How to cite this article:** Geng, Y. *et al*. Systemic Immune-Inflammation Index Predicts Prognosis of Patients with Esophageal Squamous Cell Carcinoma: A Propensity Score-matched Analysis. *Sci. Rep.* **6**, 39482; doi: 10.1038/srep39482 (2016).

**Publisher's note:** Springer Nature remains neutral with regard to jurisdictional claims in published maps and institutional affiliations.

## Figures and Tables

**Table 1:** Baseline characteristics for patients with SII ≤ 307 versus SII > 307 before and after propensity matching.

Clinical parameter	Unmatched (complete) dataset				Matched (1:2) dataset			
	SII ≤ 307 (253)	SII > 307 (663)	χ^2^	*P*	SII ≤ 307 (253)	SII > 307 (506)	χ^2^	*P*
Sex			5.24	0.022^*^			2.33	0.127
Male	179	517			179	384		
Female	74	146			74	122		
Age			0.10	0.921			0.17	0.681
≤60	125	330			125	242		
>60	128	333			128	264		
Histological grade			12.34	0.002^*^			1.50	0.472
Well differentiated	23	23			23	39		
Moderately differentiated	116	334			116	255		
Poorly or not differentiated	114	306			114	212		
Tumor location			3.90	0.142			3.08	0.214
Upper	22	35			22	28		
Middle	164	435			164	329		
Lower	67	193			67	149		
T stage			23.74	<0.001^*^			5.70	0.127
0-T1	83	125			83	125		
T2	64	161			64	149		
T3	101	363			101	222		
T4	5	14			5	10		
Examined lymph nodes			2.61	0.271			2.24	0.326
≤5	58	141			58	111		
6–15	148	366			148	277		
>15	47	156			47	118		
N stage			9.96	0.019^*^			3.65	0.302
N0	150	322			150	272		
N1	62	208			62	156		
N2	28	104			28	57		
N3	13	29			13	21		
7^th^ AJCC stage			27.94	<0.001^*^			4.44	0.109
0-I	74	94			74	114		
II	97	298			97	223		
III	82	271			82	169		

SII: systemic immune-inflammation index; AJCC: American Joint Committee on Cancer.

**Table 2:** Relationship between NLR, PLR or MLR and clinicopathological characteristics of patients with esophageal squamous cell carcinoma.

Clinical parameter	NLR				PLR				MLR			
	≤1.7 (290)	>1.7 (626)	χ^2^	*P*	≤120 (458)	>120 (458)	χ^2^	*P*	≤0.28 (496)	>0.28 (420)	χ^2^	*P*
Sex			15.44	<0.001^*^			0.38	0.536			52.93	<0.001^*^
Male	193	503			344	120			330	366		
Female	97	123			114	106			166	54		
Age			0.33	0.565			0.35	0.552			2.80	0.094
≤60	140	315			223	232			259	196		
>60	150	311			235	226			237	224		
Histological grade			8.04	0.018^*^			3.32	0.190			5.82	0.054
Well differentiated	23	23			29	17			32	14		
Moderately differentiated	143	307			223	227			248	202		
Poorly or not differentiated	124	296			206	214			216	204		
Tumor location			8.85	0.012^*^			0.54	0.764			5.90	0.052
Upper	28	29			28	29			38	19		
Middle	186	413			295	304			329	270		
Lower	76	184			135	125			129	131		
T stage			22.21	<0.001^*^			6.67	0.083			14.56	0.002^*^
0-T1	90	118			118	90			133	75		
T2	77	148			116	109			126	99		
T3	118	346			216	248			230	234		
T4	5	14			8	11			7	12		
Examined lymph nodes			0.24	0.888			0.02	0.991			1.42	0.491
≤5	63	136			99	100			107	92		
6–15	160	354			258	256			286	228		
>15	67	136			101	102			103	100		
N stage			10.04	0.003^*^			5.15	0.161			2.95	0.399
N0	171	301			247	225			267	205		
N1	72	198			131	139			139	131		
N2	30	102			56	76			66	66		
N3	17	25			24	18			24	18		
7^th^ AJCC TNM stage			26.39	<0.001^*^			8.43	0.015^*^			26.39	<0.001^*^
0-I	81	87			101	67			81	87		
II	114	281			188	207			114	281		
III	95	258			169	184			95	258		
SII			304.85	<0.001^*^			229.49	<0.001^*^			84.56	<0.001^*^
≤307	190	63			229	24			199	54		
>307	100	563			229	434			297	366		
PLR			107.56	<0.001^*^							81.33	<0.001^*^
≤120	218	240							316	142		
>120	72	386							180	278		
NLR			—	—			107.56	<0.001^*^			160.87	<0.001^*^
≤1.7	—	—			218	72			246	44		
>1.7	—	—			240	386			250	376		
MLR			160.87	<0.001^*^			81.33	<0.001^*^			—	—
≤0.28	246	250			316	180			—	—		
>0.28	44	376			142	278			—	—		

SII: systemic immune-inflammation index; PLR: platelet lymphocyte ratio; NLR: neutrophil lymphocyte ratio; MLR: monocyte lymphocyte ratio; AJCC: American Joint Committee on Cancer.

**Table 3:** Univariate and multivariate cox regression analyses for overall survival in patients with esophageal squamous cell carcinoma (unmatched complete datasets).

Variables	Univariate analysis		Multivariate analysis	
	HR (95%CI)	*P* value	HR (95%CI)	*P* value
Sex				
Male vs. Female	1.39 (1.12–1.73)	0.003^*^	1.15 (0.93–1.44)	0.206^a^
Age				
≤60 years vs. >60 years	1.12 (0.94–1.33)	0.207		
Histological grade		<0.001^*^		<0.001^*a^
Well differentiated	Ref.	—	Ref.	
Moderately differentiated	3.96 (1.87–8.40)	<0.001	2.29 (1.06–1.49)	0.034
Poorly or not differentiated	6.23 (2.94–13.20)	<0.001	3.20 (1.49–6.86)	0.003
Tumor location		0.708		
Upper	Ref.			
Middle	1.04 (0.72–1.50)	0.855		
Lower	0.95 (0.64–1.41)	0.802		
T stage		<0.001^*^		<0.001^*a^
0-T1	Ref.	—	Ref.	
T2	1.74 (1.28–2.38)	<0.001	1.38 (1.01–1.90)	0.045
T3	3.30 (2.52–4.31)	<0.001	2.08 (1.48–2.94)	<0.001
T4	4.75 (2.71–8.35)	<0.001	3.38 (1.79–6.40)	<0.001
Examined lymph nodes		0.158		
≤5	Ref.	—		
6–15	0.96 (0.77–1.19)	0.679		
>15	1.18 (0.91–1.52)	0.208		
N stage		<0.001^*^		<0.001^*a^
N0	Ref.		Ref.	
N1	2.13 (1.73–2.62)	<0.001	1.66 (1.34–2.06)	<0.001
N2	3.82 (2.99–4.87)	<0.001	2.68 (2.08–3.46)	<0.001
N3	5.45 (3.83–7.77)	<0.001	4.23 (2.95–6.05)	<0.001
SII				
>307 vs. ≤307	1.44 (1.17–1.77)	0.001^*^	1.24 (1.01–1.53)	0.042^*a^
PLR				
>120 vs. ≤120	1.23 (1.03–1.47)	0.020^*^	1.18 (0.99–1.40)	0.070^b^
NLR				
>1.7 vs. ≤1.7	1.35 (1.11–1.65)	0.002^*^	1.18 (0.97–1.44)	0.107^c^
MLR				
>0.28 vs. ≤0.28	1.26 (1.06–1.50)	0.010^*^	1.12 (0.94–1.34)	0.220^d^

SII: systemic immune-inflammation index; PLR: platelet lymphocyte ratio; NLR: neutrophil lymphocyte ratio; MLR: monocyte lymphocyte ratio; HR: hazard ratio; CI: confidence interval; Ref: reference. ^a^The variables (sex, histological grade, T stage, N stage and SII) were tested in a multivariate analysis. ^b^The variables (sex, histological grade, T stage, N stage and PLR) were tested in a multivariate analysis. ^c^The variables (sex, histological grade, T stage, N stage and NLR) were tested in a multivariate analysis. ^d^The variables (sex, histological grade, T stage, N stage and MLR) were tested in a multivariate analysis.

**Table 4:** Univariate and multivariate cox regression analyses for overall survival in patients with esophageal squamous cell carcinoma (matched datasets, 1:2).

Variables	Univariate analysis		Multivariate analysis	
	HR (95%CI)	*P* value	HR (95%CI)	*P* value
Sex				
Male vs. Female	1.29 (1.02–1.63)	0.036^*^	1.12 (0.88–1.43)	0.344
Age				
≤60 years vs. >60 years	1.14 (0.93–1.38)	0.207		
Histological grade		<0.001^*^		<0.001^*^
Well differentiated	Ref.	—	Ref.	
Moderately differentiated	3.81 (1.79–8.10)	0.001	2.10 (0.98–4.53)	0.058
Poorly or not differentiated	5.90 (2.77–12.53)	<0.001	3.00 (1.39–6.45)	0.005
Tumor location		0.540		
Upper	Ref.			
Middle	0.89 (0.60–1.32)	0.560		
Lower	0.81 (0.53–1.23)	0.319		
T stage		<0.001^*^		<0.001^*^
0-T1	Ref.	—	Ref.	
T2	1.69 (1.22–2.29)	0.001	1.33 (0.97–1.84)	0.079
T3	3.33 (2.52–4.38)	<0.001	2.11 (1.58–2.83)	<0.001
T4	4.08 (2.15–7.75)	<0.001	2.44 (1.27–4.69)	0.007
Examined lymph nodes		0.074		
≤5	Ref.	—		
6–15	0.93 (0.73–1.18)	0.549		
>15	1.28 (0.96–1.70)	0.087		
N stage		<0.001^*^		<0.001^*^
N0	Ref.		Ref.	
N1	2.43 (1.93–3.06)	<0.001	1.78 (1.40–2.28)	<0.001
N2	4.39 (3.28–5.87)	<0.001	3.12 (2.30–4.23)	<0.001
N3	6.89 (4.66–10.21)	<0.001	5.63 (3.77–8.40)	<0.001
SII				
>307 vs. ≤307	1.31 (1.06–1.63)	0.014^*^	1.30 (1.05–1.62)	0.018^* a^
PLR				
>120 vs. ≤120	1.17 (0.96–1.43)	0.112		
NLR				
>1.7 vs. ≤1.7	1.30 (1.05–1.61)	0.016^*^	1.20 (0.97–1.49)	0.093^b^
MLR				
>0.28 vs. ≤0.28	1.19 (0.98–1.45)	0.080		

SII: systemic immune-inflammation index; HR: hazard ratio; CI: confidence interval; Ref: reference. ^a^The variables (sex, histological grade, T stage, N stage and SII) were tested in a multivariate analysis. ^b^The variables (sex, histological grade, T stage, N stage and NLR) were tested in a multivariate analysis.
